# The application of innovative joint control strategies in the synergistic optimization of enterprise procurement, production, rework and quality control

**DOI:** 10.1371/journal.pone.0349198

**Published:** 2026-05-11

**Authors:** Qingyu Hong, Jiping Qi, Hao Yin, Jie Gu, Hong Shen, Jihua Fan

**Affiliations:** 1 School of Electrical and Information Engineering, Jiangsu University of Science and Technology, Zhangjiagang, China; 2 Suzhou Institute of Technology, Jiangsu University of Science and Technology, Zhangjiagang, China; University of Georgia, UNITED STATES OF AMERICA

## Abstract

This study proposes an innovative joint control strategy for enterprise collaborative optimization in procurement, production, rework, and quality control in mechanical component manufacturing enterprises. A comprehensive model unifies supplier selection, rework decisions, and quality sampling is developed, thereby addressing the fragmentation of single-stage optimization in existing studies. In procurement, a confidence-interval-based model screens suppliers, with sample size determined by binomial distribution, factoring in two types of error risks to optimize costs. A cost-benefit model for production evaluates rework feasibility. For quality control, a fraction-f sampling plan balances inspection costs and product quality. The model is solved and simulated using MATLAB, and sensitivity analysis is conducted. The analysis reveals that the spare-part defect rate significantly impacts total profit, stressing procurement quality control. The defect rate of semi-finished products has minimal impact, indicating effective controls. An increase in the finished-product defect rate raises costs and reduces profits. These results demonstrate the effectiveness of the proposed joint control strategy in optimizing the cost-quality balance.

## 1. Introduction

In the context of globalisation, fierce market competition poses significant challenges to enterprises, especially in mechanical component manufacturing enterprises, where spare-part precision, such as gear shaft dimensional accuracy, and assembly consistency directly determine product reliability. For the sustainable development of such enterprises, the synergy of multiple elements such as cost control, inventory optimisation, and balancing machine workload is crucial [1]. The production and operation of enterprises is a complex system engineering, with procurement, production, rework, and quality control interwoven and influencing each other, jointly affecting product quality, cost, and ultimately the enterprise’s profit [2]. However, multi-stage production processes are very complex systems, and any change in one part may affect the entire system [3]. In today’s market environment, where consumer expectations for product quality continue to rise, quality has become a key competitive factor for enterprises to stand out [4].

In modern industrial systems, reliability and quality issues are prominent, and quality inspection errors cannot be ignored, involving factors such as cost, customer satisfaction, regulatory compliance, and process improvement [5]. Quality inspection errors are mainly divided into Type I and Type II errors [6]. Type I error refers to the scenario in which high-quality products are incorrectly classified as nonconforming and consequently rejected, commonly referred to as “producer risk.” Conversely, Type II error occurs when defective products are mistakenly identified as conforming and therefore accepted, known as “consumer risk,” which has significant operational impacts in mechanical component manufacturing: for example, a Type II error in bearing or gear procurement may necessitate semi-finished product disassembly and rework, increasing production costs [7].

Current research has focused on adopting comprehensive approaches to achieve coordinated control of production, quality, and maintenance for deteriorating industrial systems [8], including automotive component manufacturing or electronics production, but rarely target mechanical component manufacturing enterprises, where equipment deterioration and strict assembly precision requirements introduce unique operational complexities. Under this circumstance, how to achieve coordinated optimization of procurement, production, rework and quality control while ensuring a specific confidence level, accurately determine the optimal suppliers and production strategies, and thereby maximize enterprise profits, has become a core issue that the industry urgently needs to solve.

To effectively address these challenges, both the academic and business communities have been actively exploring and have achieved a series of research results. In terms of supplier selection, Mahmoudi and Javed [9] pointed out that the traditional supplier selection framework has limitations, such as ignoring standard screening and difficulty in measuring the reliability of selected suppliers, and proposed a new confidence level measurement system using the Order Preference by Similarity to Ideal Solution (OPA) method and Kendall’s W. Additionally, Dinh et al. [10] addressed the transformation needs of Vietnamese textile and garment enterprises through association rule mining, discovering that enterprises consider corporate social responsibility (CSR) as the primary criterion for supplier selection, followed by cost, quality, and delivery. However, these studies focus on non-mechanical sectors (textiles, general manufacturing) and overlook critical factors for mechanical component supplier selection—such as integrating statistical sampling for defect rate control and Type I/II error risk assessment [11].

In the field of warranty and maintenance, Safaei and Taghipour [12] proposed an optimal preventive maintenance strategy for repairable mechanical components such as gears and bearings under a warranty strategy combining fully renewable free replacement warranty (FRW) and proportional warranty (PRW), determining the optimal replacement time by constructing a long-term expected cost rate model. However, this strategy does not account for the unique wear characteristics of mechanical components such as gear tooth abrasion. Shafiee and Chukova [13] found through a comprehensive literature review that warranty and maintenance are closely related, and optimizing maintenance strategies can reduce warranty service costs, providing a clear classification framework for subsequent research. Karki and Porras [14] focused on the role of digitalization in maintenance services, stating that digitalization can improve maintenance service efficiency and have a positive impact on sustainable development. Yet these studies lack integration with procurement and quality control decisions in a unified optimization framework for mechanical component manufacturing.

In the field of quality inspection, traditional attribute sampling inspection plans consider the proportion of non-conforming mechanical components as the evaluation criterion, including the screening attribute sampling inspection plans defined under LTPD and AOQL schemes [15]. However, these plans are less suitable for high-precision mechanical components where minor defects can lead to cascading failures in subsequent assembly. Various improved sampling plans have emerged, such as MRGSP, which combines historical batch quality records with the repeated sampling mechanism [16], yet MRGSP rarely incorporates dynamic defect rate changes caused by tool wear, limiting its adaptability to real-time production environments. In complex production decision-making scenarios, fuzzy multi-attribute decision-making is often used to handle the fuzziness and incompleteness of information [17], with extensions to intuitionistic fuzzy programming for optimizing sample allocations in stratified surveys relevant to quality estimation under uncertainty [18]. Mathematical programming models are also significant in optimizing production decisions: multi-objective programming (MOP) models can balance multiple objectives such as material efficiency, production precision, and profit [19], and has been further adapted to mechanical component scenarios, as demonstrated in hardware production planning under intuitionistic and neutrosophic fuzzy environments [20], case studies for optimal multi-product hardware output [21], lock industry applications balancing production costs and capacities [22], and intuitionistic fuzzy supply chain logistics for multi-product networks [23]; while linear programming [24] and integer programming models [25] can also play a role in other mechanical component production scenarios, such as resource allocation and equipment purchase quantity determination, yet these models often rely on static parameters that do not reflect the variability of mechanical component production, limiting their ability to assist mechanical component manufacturing enterprises in dynamic quality-control decisions.

However, these traditional methods have notable drawbacks. Traditional statistical sampling inspection methods are susceptible to sampling errors, and the quality characteristics of samples may not accurately reflect the overall situation, potentially leading to misjudgment of product quality [26]. Mathematical programming models also face challenges as their constraints and parameters are often based on assumptions or historical data [27], while the actual production environment is complex and variable [20–23]. Moreover, traditional models often consider procurement, production, rework, and quality control separately, failing to fully reflect the close connections among these links.This fragmented approach constitutes a critical research gap: existing studies lack an integrated framework that simultaneously optimizes supplier selection with statistical confidence intervals, production-rework decisions with cost-benefit analysis, and quality control with adaptive sampling strategies while explicitly accounting for Type I/II inspection errors.

This paper addresses these challenges and proposes an innovative joint control strategy that views procurement, production, rework, and quality control as an organic whole, aiming to maximise mechanical component manufacturing enterprise profits. The core idea is to incorporate the costs and benefits of each link into a unified mathematical framework, with the objective function being the maximisation of total profit. The procurement control strategy ensures the selection of high-quality suppliers by constructing a supplier selection model based on confidence intervals and optimising the number of inspections; the production and rework control strategy formulates scientific rework and disassembly decisions based on cost-benefit comparison, reducing production costs and ensuring mechanical component quality; the quality control strategy adopts a fraction-f sampling plan, balancing inspection costs and average outgoing quality to ensure that mechanical component quality meets customer requirements. To verify the effectiveness of this strategy, a comprehensive mathematical model is established and solved using MATLAB. The model is validated through an empirical case study utilizing production data from a publicly listed precision gear manufacturer, accompanied by multicollinearity diagnostics to ensure statistical robustness. Furthermore, sensitivity analysis is conducted to examine the influence of key parameters on total profit. The research results show that the joint control strategy proposed in this paper can effectively balance procurement, production, rework, and quality costs, significantly increasing enterprise profits.

## 2. System Description

The purpose of this study is to develop and validate a joint control strategy for procurement, production, rework, and quality control tailored to mechanical component manufacturing enterprises under a certain confidence level. It aims to identify optimal mechanical spare part suppliers and determine optimal production strategies for such enterprises, so as to ultimately maximize profits. This section covers three core components: definitions of key symbols aligned with mechanical component manufacturing scenarios, descriptions of the operational system, and analysis of system errors.

### 2.1. Symbol list

The issues under review rely on the following notes:

Core decision variables

$x_{\text{1}}$ ~ $x_{n}$: Indicates whether *n* parts are inspected.

$x_{n+1}$ ~ $x_{n+m}$: Indicates whether *m* kinds of semi-finished products are inspected

$x_{n+m+1}$: Indicates whether the finished product is inspected.

$x_{n+m+2}$ ~ $x_{n+2m+1}$: Indicates whether *m* semi-finished products are disassembled.

$x_{n+2m+2}$: Indicates whether the finished product is disassembled.

f: The proportion of parts tested.

Quality-related parameters

α: The rate of the first type of error in the detection.

β: The rate of the second type of error in the detection.

P: Rate of defective products detected.

$p_{\text{0}}$: Assumed nominal defective rate.

$p_{\text{1}}$: The rate of defective products that companies want to be able to detect.

$R_{\text{1}}$ ~ $R_{n}$: Indicates the rate of defective parts after updating.

$R_{n+1}$ ~ $R_{n+m}$: Represents the rate of defective semi-finished products after updating.

$R_{n+m+1}$: Represents the rate of defective finished products after updating.

AOQ: The average factory quality reaching customers is also the proportion of defective products when finished products enter the market.

AOQmax: The maximum limit of the average output quality is set by the customer and also the maximum limit of the proportion of defective products when the finished products enter the market f(p): The prior distribution density function of *p*.

Cost and benefit parameters

$C_{l}$: Spare parts cost.

$C_{b}$: The cost of semi-finished products.

$C_{c}$: Cost of finished goods.

$C_{ci}$: Defective cost.

$C_{bcx}$: Cost of disassembly of semi-finished products.

$C_{ccx}$: Cost of disassembly of finished products.

$J_{l}$: The purchase price of spare parts.

$J_{zz}$: Unit price of assembly.

$K_{l}$: Unit price of parts inspection.

$K_{b}$: Unit price of semi-finished product inspection.

$J_{zz}$: Unit price of assembly.

$K_{c}$: Unit price of finished product inspection.

$J_{s}$: Market price.

$J_{h}$: Replacement unit price.

$J_{bcx}$: Unit price of disassembly of semi-finished products.

$J_{ccx}$: Disassembly cost.

$P_{s}$: Revenue from sales.

$p_{cc}$: Profit from dismantling the finished product.

$p_{bc}$: Profit from disassembling half-finished products.

$p_{z}$: Gross profit.

Quantity parameter

n: Sample size.

N: Population quantity.

$N_{\text{1}}$: Total purchases of spare parts.

$N_{\text{2}}$: Quantity of defective goods

$N_{\text{3}}$: Number of semi-finished products.

$N_{li}$: Type *i* number of parts available for synthesis.

$N_{bi}$: The resultant amount of the i semi-finished product.

$N_{c}$: The resultant amount of the finished product.

p(X=k): In the sample, the defect rate of *k* times was observed.

$p_{a}\left(p|f\right)$: Acceptance probability

Statistical auxiliary parameters

$\left(\begin{array}{c} @l@n\\ k \end{array}\right)$: Representation combination number.

c: The number of acceptances in the sampling plan

$Z_{\alpha }$: The critical value of the normal distribution corresponding to the significance level.

$Z_{\beta }$: Test the critical value of the normal distribution corresponding to the force.

FPC: Finite population correction coefficient.

Z: The correction factor represents the proportion of semi-finished products that meet certain conditions.

### 2.2. Product life cycle system under the joint control strategy

**Fig 1:**
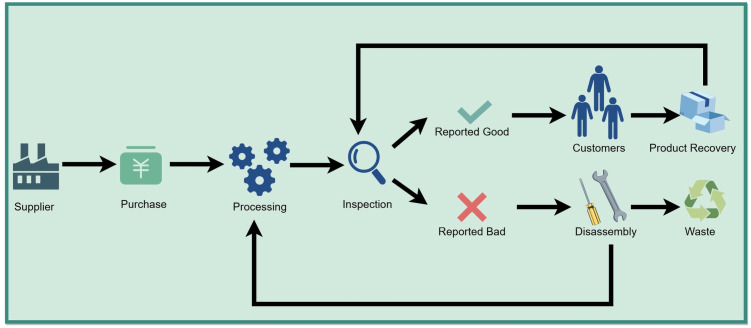
Product cycle diagram.

**Fig 1** details the life cycle of a mechanical component product, which begins with the initial “procurement” phase. In this phase, suppliers are selected based on their maximum supply capacity of mechanical spare parts and the defect rate of these parts. Next is the “processing” stage, where mechanical spare parts are processed into semi-finished mechanical components. Subsequently, the life cycle proceeds to the “inspection” phase, where the manufactured semi-finished mechanical components are inspected to ensure compliance with preset quality standards for mechanical components. Unqualified semi-finished mechanical components identified during inspection are addressed before advancing to the “sales” phase—either via rework (for repairable defects) or return to the upstream production process. For products that cannot be repaired, the “recycling” phase involves disassembling them to recover and reprocess usable mechanical spare parts, while those that are completely damaged can only be discarded. The objective of this process is to ensure that the final mechanical products delivered to customers meet their requirements for both quantity and quality specifications.

The occurrence of failure is related to factors such as equipment maintenance, usage patterns and equipment design. These factors can be unified and abstracted as a defective rate problem. To reduce the adverse impact of faults, the system adopts a disassembly strategy. When the mechanical product is detected as unqualified, the system will immediately determine whether to disassemble according to the cost-benefit analysis, and then return the disassembled mechanical parts to the production station.

Due to the lack of comprehensive inspection of the mechanical product and the existence of inspection errors, defective mechanical products will inevitably enter the market, and customers may thus receive defective mechanical components, including those undetected during inspection and those missed due to inspection errors, which can lead to serious consequences. The resulting losses are collectively referred to as “recovery” losses, which correspond to the average outgoing quality (AOQ) and the customer’s post-sales costs. In order to reduce such losses, this paper determines whether the recovered products need to be repaired and disassembled based on the overall income, and then sends the disassembled parts back to the production station.

The objective of this paper is to propose a joint procurement, production, rework, and quality control policy, and then establish an effective model to minimise costs and increase profits. This profit is calculated as sales revenue plus disassembly revenue minus production costs, inspection costs, and recovery costs. The optimal solution lies in achieving an appropriate balance.

### 2.3. System errors

System errors in quality control for mechanical component manufacturing are often overlooked and lead to decision-making errors. This paper focuses on the system errors of “procurement” and “production”. “Procurement” system errors refer to two types of errors in inspection, namely false rejection of good products (producer risk) and false acceptance of defective products (consumer risk), which either increase unnecessary procurement costs (Type I error) or raise the risk of defective parts entering production (Type II error). “Production” system errors include source errors and chain errors, which affect production line efficiency. These errors are abstracted as a random defective rate.

**Fig 2:**
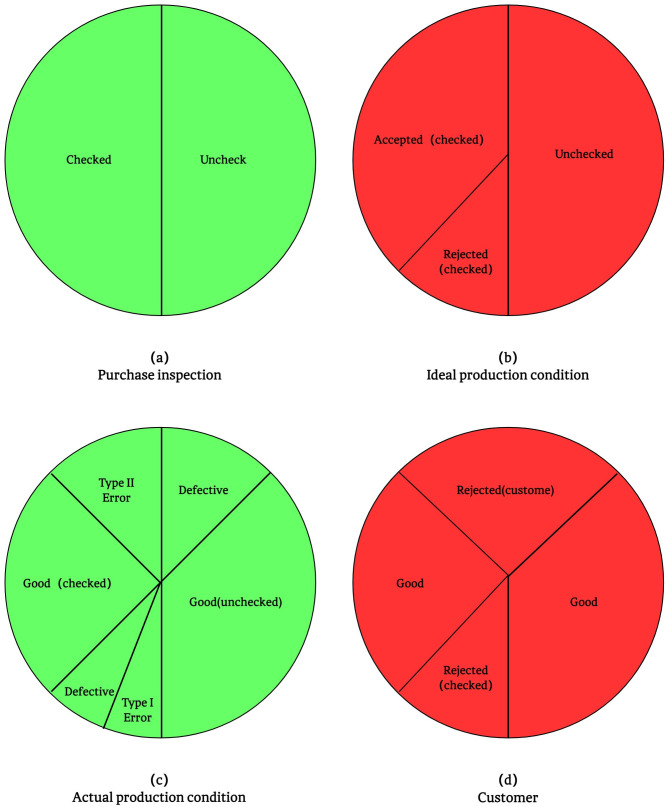
Production and marketing structure diagram caused by the first and second type of inspection errors.

Fig 2 summarises the impact of inspection and system errors in the mechanical component production process. Details from (a) to (d) will be explained below.

In the “procurement” step (a), a sample test is carried out on the product, and the sample proportion is set to *f*, while the remaining 1-*f* proportion of the product does not need to be tested. After completion of the testing process (b), some products are rejected for nonconformity with the ratio f((1−p)α)), while the proportion of qualified and accepted products is f((1−p)(1−α)). However, since the inspection process is not absolutely accurate (c), the first type of error may occur, namely, the erroneous rejection of actually qualified products. In this case, some of the rejected products are actually qualified, but they are mistakenly rejected due to inspection errors, which undoubtedly causes a certain degree of loss.

Entering the “processing” phase, the inspected parts are used in the manufacture of the product. In the process, of the rejected parts accounted for f((1−p)(1−α)β), and be part of the proportion of f((1−p)(1−α)(1−β)). It is worth noting that a second type of error can occur in this process, namely, the mistaken acceptance of products that are actually substandard. As a result, some of the accepted products are actually defective, but due to inspection errors, they flow into the subsequent production process.

Eventually, all products, whether tested or not, will be sold to customers (d). It should be emphasised that due to the errors in the inspection process and the possible defects in the production process itself, the final product batch can be defined by two key quality metrics: the proportion of effectively conforming products, which quantifies valid qualified items in the batch, is calculated via Eq. 1; the average proportion of defective products delivered to customers, which reflects the post-delivery defect risk, is determined by Eq. 2.


δ=1−f·[(1−p)·α+p·(1−β)] (1)

ρ=(1−f)·p+f·p·β (2)

## 3. Control policies

The proposed policy is based on the integration of procurement, production, rework, and quality control for mechanical component manufacturing enterprises. The following is a detailed description of the three policy dimensions of procurement, production and rework, and quality control, as well as the related decision variables.

### 3.1. Procurement control strategy

In supply chain management for mechanical component manufacturing enterprises, requirements for the reliability of supplier selection are increasingly stringent—enterprises often set a specific reliability level r and exclude suppliers that fail to meet this reliability level. Eq. 3 shows that:

i) If the upper limit of the estimated confidence interval is less than or equal to the nominal defective rate, it can be considered that the defective rate of mechanical spare parts does not exceed the nominal value, and the enterprise can accept the batch of mechanical spare parts.
ii) If the lower limit of the confidence interval is greater than the nominal defective rate, it can be considered that the defective rate of mechanical spare parts exceeds the nominal value, and the enterprise should reject the batch of mechanical spare parts.

$$\left\{ \begin{array}{c} @l@p+Z_{\alpha }\cdot \sqrt{\frac{p\left(1-p\right)}{n}}\leq p_{\text{0}}\\ p-Z_{\alpha }\cdot \sqrt{\frac{p\left(1-p\right)}{n}}\geq p_{\text{0}} \end{array}\right.$$ (3)

Corresponding to the next stage, the best supplier is selected from the selected suppliers, and the number of tests is required to be less. According to the definition of Eq. 4, it indicates that the mechanical component manufacturing enterprise determines *w*_1_ and *w*_2_ according to the importance of the test cost and test effect. If the enterprise considers the test effect to be above, *w*_2_ should be larger; if the enterprise believes that reducing the cost of testing is important, *w*_1_ should be larger. The final value of n is actually a trade-off between the cost of detection and the effectiveness of detection.


$$g\left(n\right)=w_{\text{1}}cn-w_{\text{2}}\underset{p\in \theta_{\text{1}}}{\int }f\left(p\right)\cdot {\text{Z}}_{\beta }\cdot dp$$ (4)

### 3.2. Production and rework control strategy

The total profit maximization objective of the proposed integrated model inherently addresses production line balancing in mechanical component manufacturing, and embeds core modern lean management principles—including takt time optimization and value stream analysis—into the multi-link decision-making framework rather than treating them as independent factors. For the production system with system errors and variable defect rates, reducing production-related costs requires scientific disassembly and rework decisions for unqualified mechanical components, and the lean-oriented decision logic is reflected in the cost-benefit comparison of each production link to eliminate waste and optimize process efficiency. According to Eq. 5 and Eq. 6, it indicates that if the benefit of disassembly is greater than the cost of inspection and recovery, the inspection is carried out, and the reverse is not carried out. If the disassembly income is greater than the production cost, the disassembly is carried out, and the reverse is not carried out.


$$\text{Detection}\left\{ \begin{array}{c} @l@{\text{Yes\textasciitilde{}\textasciitilde{}if p}}_{cc}>C_{l}+C_{ci}\\ {\text{NO\textasciitilde{} if p}}_{cc}<C_{l}+C_{ci} \end{array}\right.$$ (5)

$$\text{Rework}\left\{ \begin{array}{c} @l@{\text{Yes \textasciitilde{}\textasciitilde{}if p}}_{cc}\;>C_{bcx}+C_{ccx}+C_{c}\\ {\text{NO \textbackslash{},\textbackslash{},\textasciitilde{}if p}}_{cc}\;>C_{bcx}+C_{ccx}+C_{c} \end{array}\right.$$ (6)

### 3.3. Quality control policy

For product quality control in mechanical component manufacturing, a sampling plan based on the optimal sample size is more practical than the 100% inspection strategy. By integrating a multi-criteria evaluation system into the sampling framework, the conventional single-sampling method can be extended to accommodate multi-dimensional scenarios—specifically, adopting the fraction-f sampling plan for quality assessment proves more economical and scientifically sound for mechanical components. During the inspection process, direct costs are incurred, necessitating a quantitative assessment of the average outgoing quality (AOQ). Therefore, accurately determining the optimal inspection quantity is crucial. By integrating a multi-criteria evaluation system into the sampling plan, the conventional single-sampling method can be extended to accommodate multi-dimensional scenarios. Specifically, each criterion must undergo an independent test, and a product lot is accepted only when all tests are passed, meeting the predefined overall nonconformance rate and risk tolerance level.

Building upon this foundation, a dynamic sampling ratio *f* is introduced in the sampling procedures during the mechanical spare part procurement phase. This approach allows for flexible regulation of the inspection process, achieving a precise balance between inspection costs and quality assurance for mechanical components. Similar to quality criterion must correspond to an independent test, and a product lot is accepted only when all tests are passed, meeting the predefined overall nonconformity rate and risk level.

Building upon this framework, how traditional sampling plans are optimized using the operating characteristic (OC) curve and the range of average outgoing quality (AOQ), a parametric optimization model for the sampling ratio *f* can significantly reduce a dynamic sampling ratio *f* is introduced in sampling operations at the procurement stage. This approach allows for flexible regulation of the sampling process, achieving a precise balance between inspection costs and quality assurance. Similar to optimizing overall inspection costs across the entire process while maintaining the desired confidence level in product quality.

Average Output Quality (AOQ) Eq. 7


$$AOQ=\frac{\left(1-f\right)\cdot p+f\cdot p\cdot \beta }{1-f\cdot \left[\left(1-f\right)\cdot p+f\cdot p\cdot \beta \right]}$$ (7)

The optimal policy should then ensure that the AOQ does not exceed the maximum average output quality (AOQmax) defined by the customer. However, if the detection cost is greater than the recovery cost caused by non-detection, you can choose not to inspect.

### 3.4. OC Curve and Type I/II error for fraction-f sampling

To provide theoretical justification for the proposed fraction-f attribute sampling plan and explicitly quantify inspection errors, this section presents an operating characteristic (OC) curve analysis. For a single-sampling plan with sample size n and acceptance number c, the probability of accepting a lot with true defect rate p is theoretically derived from the binomial distribution probability mass function. Relevant theoretical foundations for the OC function under fraction-f attribute inspection have been reported in recent research on novel acceptance sampling plans for lot disposition [28]. Under the fraction-f sampling scheme, the OC function is formally established and expressed as shown below, where the derivation fully reflects the binomial distribution properties of pass–fail inspection outcomes.


$$p_{a}\left(p|f\right)=\sum_{k=0}^{c}\left(\begin{array}{c} @l@n\\ k \end{array}\right)p^{k}{\left(1-p\right)}^{n-k}$$ (8)

where p_a_ denotes the acceptance probability, f is the sampling fraction, n is the lot size, k represents observed defectives, and c is the acceptance number.

Based on the OC curve formulation in Eq. 8, Type I error and Type II error are explicitly modeled. At the acceptable quality level p_0_, Type I error is defined as $\alpha \;=\;1\;-\;p_{a}\left(p_{\text{0}}|f\right)$; at the lot tolerance percent defective p_1_, Type II error is $\beta \;=p_{a}\left(p_{\text{1}}|f\right)$. These error terms are incorporated into the integrated objective function (Eq. 40) through the defective product cost term, enabling joint optimization of inspection intensity and overall system profit.

It should be noted that as f decreases, the OC curve becomes flatter, resulting in elevated α and β values. The fraction-f sampling plan is therefore designed for general mechanical components where cost-quality trade-offs are prioritized. For safety-critical parts, fractional sampling is generally inadmissible, and 100% inspection or zero-acceptance plans compliant with AS9100 or IATF 16949 standards are typically required.

## 4. Parameter optimization of control strategy

This section provides mathematical formulas for the problems studied as described in Section 3. Relevant cost and mathematical models will be developed to optimise the parameters of the proposed integrated policy.

### 4.1. Update process

**Fig 3:**
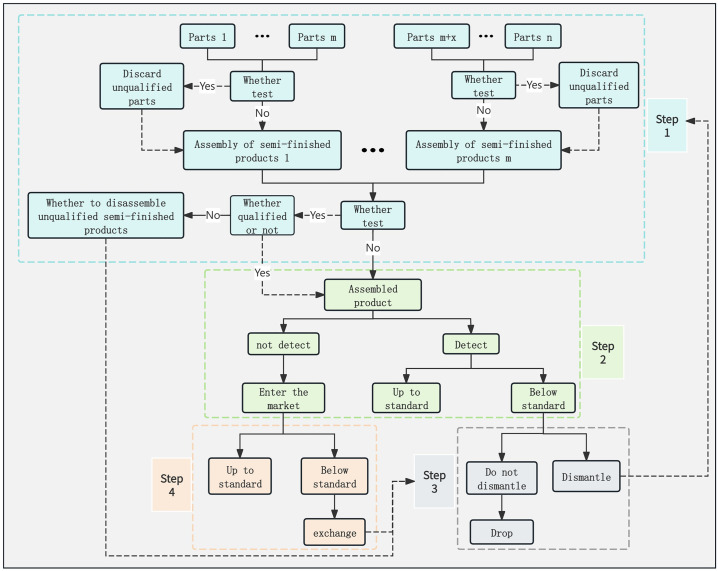
Decision flow chart.

Processing mechanical parts into finished products requires multiple steps. Fig 3 shows the production flow of the enterprise in the production process of the product. It is mainly divided into four steps. Step 1: Each mechanical part (from Part 1 to Part n) is first evaluated to determine if testing is needed, if the test finds that the part is unqualified, these parts will be directly discarded to ensure the quality of the subsequent production process. For the parts that pass the inspection, they will be used for assembly to form a preliminary semi-finished product. Next, the enterprise determines whether to inspect the semi-finished products. If the semi-finished products are found to be unqualified, these semi-finished products will be judged whether to disassemble to ensure the quality of the subsequent production process. For the semi-finished products that pass the inspection, they will be used for assembly to form the finished product. Step 2: Once finished products are manufactured, the final inspection is required. In this step, the enterprise may choose to allow finished products to enter the market without testing, or be tested to ensure quality. If the test result is qualified, the finished product will be allowed to enter the market. If it fails, it needs to be disassembled or otherwise disposed of. Step 3 determines whether to disassemble semi-finished products and finished products. If disassembled, perform Step 1 for mechanical spare parts. If not, discard them directly. Step 4: For finished products entering the market, if quality problems are found through market feedback, these products will be recalled and replaced. For unqualified products, you can choose to discard them directly without disassembly, or disassemble them to recycle usable parts or materials and reduce waste. This step ensures the final quality of the product and customer satisfaction.

Fig 3 shows a production system of mechanical parts in industrial manufacturing. For the purpose of optimal efficiency, the system considers a multi-level and multi-stage production system because the production system is too complex. Through this model, the maximum profit of n mechanical parts of a finished product can be obtained under different defective rates, purchase costs, inspection costs, disassembly costs and assembly costs at different market prices. On this basis, the existence of semi-finished products is taken into account, forming a multi-level production process. Additionally, the issue of the unqualified products purchased by customers is addressed, as well as the exchange losses incurred from unconditional exchanges.The purpose of this model is to determine the optimal strategy for the multi-level, multi-stage production system, with the ultimate goal of maximizing profit.

### 4.2. Mathematical model

This section presents a mathematical model of the problem, and all the following equations are based on the production flow shown in Fig 1 and Fig 3.

In the scope of procurement quality control, it is crucial to accurately assess the defective rate of mechanical spare parts. In related studies, Al-Bossly et al. proposed the method of building a probability model based on binomial technology, which provided strong support for accurately evaluating the defective rate of mechanical spare parts [29]. With this idea in mind, this study constructs the following hypothesis testing model:

Set the original hypothesis *H*_0_: let the defective rate of mechanical parts not be greater than its nominal value; Alternative hypothesis *H*_1_: The defective rate of mechanical parts is greater than its nominal value.

Original hypothesis *H*_0_: $P\leq P_{\text{0}}$
Alternative hypothesis *H*_1_: $P>P_{\text{0}}$

According to the characteristics of binomial distribution, the necessary sample size was determined to ensure that the detection results were statistically significant. In the sampling stage, a certain number of mechanical spare parts are randomly selected for inspection, and the number of defective products is recorded. Using this data, the actual observed value of the defective rate is calculated.

Using the binomial distribution’s probability mass function (PMF), it is possible to estimate the probability of observing the current or more extreme defect rate, i.e., the *p*-value, if the null hypothesis is true. If the *p*-value is lower than the pre-set significance level (such as the common 0.05), the null hypothesis *H*_0_ is rejected and the alternative hypothesis *H*_1_ is accepted, indicating that the defective rate of mechanical parts exceeds the nominal value. Conversely, if the *p*-value is higher than the significance level, the null hypothesis *H*_0_ cannot be rejected, meaning that there is insufficient statistical evidence to support the idea that the mechanical parts defective rate exceeds the nominal value.

Probability of non-conforming products in small samples: Eq. 9 indicates that when the sample size is less than 30, it can be used to determine whether there is a significant difference between the rate of defective products in the sample and the pre-set proportion of the population.


$$p\left(X=k\right)=\left(\begin{array}{c} @l@n\\ k \end{array}\right)p^{k}{\left(1-k\right)}^{n-k}\;n<30$$ (9)

The first type of Z-test error under an infinite sample size: when Eq. 10 shows that the null hypothesis is true, a true null hypothesis is wrongly rejected, and the critical value of the normal distribution corresponding to the significance level is calculated.


$$Z_{\alpha }=\frac{p-p_{\text{0}}}{\sqrt{\frac{p_{\text{0}}\left(1-p_{\text{0}}\right)}{n}}}$$ (10)

The second type of Z-test error under infinite sample size: Eq. 11 indicates that when the null hypothesis is false, no false null hypothesis is rejected, and the normal distribution critical value corresponding to the test force is calculated.


$$Z_{\beta }=\frac{p_{\text{1}}-p_{\text{0}}}{\sqrt{\frac{p_{\text{1}}\left(1-p_{\text{1}}\right)}{n}}}$$ (11)

Sample size *n*: Eq. 12 calculates the sample size for sampling detection when the sample size is infinite.


$$n={\left(\frac{Z_{\alpha }+Z_{\beta }}{p_{\text{1}}-p_{\text{0}}}\right)}^{\text{2}}\cdot \left(p_{\text{0}}\cdot \left(1-p_{\text{0}}\right)\right)$$ (12)

The first type of error with limited sample size and large size: Eq. 13, Eq. 14 and Eq. 15 show the correction of the first type of error under the Z test by the FPC correction factor.


$$FPC=\frac{N-n}{N-1}$$ (13)

$$Z_{\alpha }=\frac{p-p_{\text{0}}}{\sqrt{\frac{p_{\text{0}}\left(1-p_{\text{0}}\right)}{n}\cdot FPC}}$$ (14)

$$Z_{\beta }=\frac{p_{\text{1}}-p_{\text{0}}}{\sqrt{\frac{p_{\text{1}}\left(1-p_{\text{1}}\right)}{n}\cdot FPC}}$$ (15)

Correction of sample number *n*: Eq. 16, Eq. 17, and Eq. 18 indicate the correction of the FPC correction factor to the sample size under limited samples.


$$n^{\text{'}}=n\cdot FPC$$ (16)

$$FPC\approx \frac{N-n}{N}$$ (17)

$$n\text{'}=\frac{n}{1+\frac{n}{N}}$$ (18)

Reliability testing is essential at multiple stages of mechanical component development, manufacturer shipment, and customer acceptance, applicable to individual parts, semi-finished products, and finished systems. Given the high time and material costs of reliability testing, a sequential probability ratio test (SPRT) approach is adopted to minimize the number of tested units and improve inspection efficiency for mechanical component manufacturing, with its specific implementation procedures and statistical decision criteria for defective rate verification detailed in Eq. 19 to Eq. 22.

Confidence interval: Eq. 19 calculates the confidence interval to estimate whether the sample defect rate is within the acceptable range of the enterprise. If the upper limit of the estimated confidence interval is less than or equal to the nominal defective rate, it can be considered that the defective rate of spare mechanical parts does not exceed the nominal value, and the enterprise can receive the batch of mechanical spare parts; if the lower limit of the confidence interval is greater than the nominal defective rate, it can be considered that the defective rate of mechanical spare parts exceeds the nominal value, and the enterprise should reject the batch of mechanical spare parts.


$$p\pm Z_{\alpha }\cdot \sqrt{\frac{p\left(1-p\right)}{n}}$$ (19)

Number of detections: Eq. 20 indicates the acceptance condition for the null hypothesis H_0_; Eq. 21 indicates the rejection condition for the null hypothesis H_0_ against the alternative hypothesis H_1_; Eq. 22 indicates the conditions for selecting to continue sampling. This sequential decision-making is based on the likelihood ratio $p\left(X=k|p_{\text{1}}\right)/p\left(X=k|p_{\text{1}}\right)$, with Type I error α and Type II error β strictly controlled in the testing process (critical values defined as A=β/1−α and B=1−β/α). Eq. 19 to Eq. 22 show that by using SPRT, the number of detections can be dynamically adjusted, and the preset decision criteria can still be met while the sample size is reduced as much as possible.


$$\frac{p\left(X=k|p_{\text{0}}\right)}{p\left(X=k|p_{\text{1}}\right)}<A$$ (20)

$$\frac{p\left(X=k|p_{\text{0}}\right)}{p\left(X=k|p_{\text{1}}\right)}>B$$ (21)

$$A\leq \frac{p\left(X=k|p_{\text{0}}\right)}{p\left(X=k|p_{\text{1}}\right)}\leq B$$ (22)

Update of defective mechanical parts rate: Eq. 23, which means that the detection results are combined with the previous empirical data (prior probability) of the enterprise to obtain a new estimate of the defective rate. By constantly adjusting the probability distribution of the defective rate, it can more accurately reflect the actual situation, so as to improve the accuracy and reliability of decision-making.


$$P\left(X=k|p\right)=\left(\begin{array}{c} @l@n\\ k \end{array}\right)p^{k}{\left(1-p\right)}^{n-k}$$ (23)

Cost of mechanical spare parts: Eq. 24 encompasses both the fixed purchase cost and the variable inspection cost.


$$C_{l}=J_{l}\cdot N_{\text{1}}+K_{l}\cdot x\cdot N_{\text{1}}$$ (24)

Cost of semi-finished products: Eq. 25 encompasses both the fixed assembly cost and the variable inspection cost.


$$C_{b}=J_{zz}\cdot N_{b}+K_{b}\cdot x_{n+i}\cdot N_{bi}\text{i}\in \left[1,m\right]$$ (25)

Cost of finished product: Eq. 26 encompasses both the fixed assembly cost and the variable inspection cost.


$$C_{c}=J_{zz}\cdot N_{c}+K_{c}\cdot x_{n+m+1}\cdot N_{c}$$ (26)

Defective product cost: Eq. 27 encompasses the fixed compensation cost, the fixed replacement cost, and the variable disassembly cost.


$$C_{ci}=J_{s}\cdot N_{\text{2}}+J_{h}\cdot N_{\text{2}}+C_{bcx}+C_{ccx}$$ (27)

Disassembly cost of semi-finished products: Eq. 28 calculates the disassembly cost of semi-finished products. If the semi-finished products fail to pass the inspection, it is also necessary to analyse whether to disassemble them.


$$C_{bcx}=J_{bcx}\cdot N_{\text{3}}\cdot x_{n+m+1+i}\text{i}\in \left[1,m\right]$$ (28)

Disassembly cost of the finished product: Eq. 29 calculate the disassembly cost of the finished product, and analyse whether to disassemble the finished product if it fails to pass the inspection.


$$C_{ccx}=J_{ccx}\cdot N_{\text{2}}\cdot x_{n+2m+2}$$ (29)

Profit from sales: Eq. 30 represents the profit from the sale of the product.


$$P_{s}=N_{c}\cdot J_{s}$$ (30)

Revenue from disassembly: Eq. 31 calculates revenue from semi-finished product disassembly, and Eq. 32 calculates revenue from finished product disassembly. In the calculation process, there are differences between the disassembly of semi-finished products and the disassembly of finished products. For semi-finished products, it is necessary to evaluate their quality, not just based on price. Therefore, a correction factor Zi is introduced, representing the proportion of semi-finished products that meet certain conditions.


$$p_{bc}=\sum_{i=1}^{n}N_{i}\cdot J_{li}$$ (31)

$$p_{cc}=\sum_{i=1}^{m}N_{i}\cdot J_{zzi}\cdot Z_{i}$$ (32)

Number of mechanical parts: Eq. 33 to Eq. 35 indicate the number of different mechanical parts.


$$N_{l1}=\left\{ \begin{array}{c} @l@N,x_{\text{1}}=0\\ \pi \left(1-R_{\text{1}}\right)\cdot N,x_{\text{1}}=1 \end{array}\right.$$ (33)

$$N_{l2}=\left\{ \begin{array}{c} @l@N,x_{\text{2}}=0\\ \pi \left(1-R_{\text{2}}\right)\cdot N,x_{\text{2}}=1 \end{array}\right.$$ (34)

$$N_{ln}=\left\{ \begin{array}{c} @l@N,x_{n}=0\\ \pi \left(1-R_{n}\right)\cdot N,x_{n}=1 \end{array}\right.$$ (35)

Synthesis of semi-finished products: Eq. 36 to Eq. 38 indicates the quantity of different semi-finished products.


$$N_{b1}=min\{N_{l1},...,N_{lx_{\text{1}}}\}$$ (36)

$$N_{b2}=min\{N_{lx_{\text{1}}},...,N_{lx_{\text{2}}}\}$$ (37)

$$N_{bm}=min\{N_{lx_{m-1}},...,N_{ln}\}$$ (38)

Composite amount of finished products: Eq. 39 indicates the number of different finished products.


$$N_{c}=min\{N_{b1},N_{b2},......N_{bn}\}$$ (39)

Total profit: Eq. 40 represents the objective function of the model. The purpose of this model is to reduce the “procurement” cost and increase the “production”.


$$p_{z}=p_{s}+p_{bc}+p_{cc}-C_{l}-C_{c}-C_{ci}-C_{b}$$ (40)

The proposed mathematical model incorporates an objective function (Eq. 40) that supports decision-making regarding inspection, disassembly, and related operations for n mechanical parts, m semi-finished products, and one finished product. Consequently, the optimal solution necessitates achieving a balance among the various parts, semi-finished products, and finished products. To address this optimization problem, two numerical programs are developed and implemented in MATLAB. The model is subsequently validated through an empirical case study utilizing production data from a precision gear manufacturer, accompanied by multicollinearity diagnostics to ensure the statistical robustness of the parameter estimates. Furthermore, sensitivity analysis is conducted to systematically examine the influence of key parameters on total profit.

## 5. Experiment and numerical calculation

This section presents the results of the innovative joint procurement-production-rework-quality control strategy proposed in Section 3 by summarizing experimental data and numerical simulation results, with a specific focus on mechanical component manufacturing enterprises. The parameter settings of the following numerical programs are based on the core experimental data presented in Table 1 and Table 2 of this manuscript. These parameters are derived from the industry research dataset released by the China Machinery Industry Federation in 2023, which was compiled through a structured field survey encompassing 15 typical mechanical component manufacturing enterprises located in the Yangtze River Delta and Pearl River Delta regions of China. Spare part purchase costs and unit inspection fees were obtained from procurement and financial records, defect rates were calculated from quality inspection logs, and assembly expenses were derived from cost accounting data. The 90% confidence level adopted for screening supplier-related data aligns with the confidence-interval-based supplier selection model in Section 3.1, thereby ensuring the reliability of input parameters for subsequent calculations. To further validate the practical applicability of the proposed model, an empirical case study is conducted using production and financial data from a publicly listed precision gear manufacturer in China. The case study parameters, including defect rates, inspection costs, and assembly expenses, were extracted from the company’s publicly disclosed annual reports and supplemented by operational data obtained through enterprise cooperation. Detailed data required for result verification, including Bayesian-updated defect rates and raw sensitivity analysis data, together with the code for reproducing numerical calculations and visualizations, are provided in Supporting Information S1 File and S2 File, respectively, to facilitate the verification and reproduction of the study’s numerical methods and results.

**Table 1:** The situations encountered by enterprises in production.

Condition	Parts 1			Parts 2			Finished product				Defective finished product	
	Defective rate	Purchase price	Inspection cost	Defective rate	Purchase price	Inspection cost	Defective rate	Assembly cost	Inspection cost	Market price	Replace-ment loss	Dismantling cost
1	10%	4	2	10%	18	3	10%	6	3	56	6	5
2	20%	4	2	20%	18	3	20%	6	3	56	6	5
3	10%	4	2	10%	18	3	10%	6	3	56	30	5
4	20%	4	1	20%	18	1	20%	6	2	56	30	5
5	10%	4	8	20%	18	1	10%	6	2	56	10	5
6	5%	4	2	5%	18	3	5%	6	3	56	10	40

**Table 2:** Situations encountered by enterprises in production.

Spare parts and accessories	Defective rate	Purchase price	Inspection cost	Semi-finished product	Defective rate	Assembly cost	Inspection cost	Dismantling cost
1	10%	2	1	1	10%	8	4	6
2	10%	8	1	2	10%	8	4	6
3	10%	12	2	3	10%	8	4	6
4	10%	2	1					
5	10%	8	1	**Finished product**	10%	8	6	10
6	10%	12	2					
7	10%	8	1		**Market price**		**Replacement loss**	
8	10%	12	2	**Finished product**	200		40	

### 5.1. Numerical Program 1

This numerical program focuses on the “spare part–finished product” two-stage production scenario, verifying how the proposed joint control strategy adjusts inspection decisions and optimizes profit under varying quality and cost conditions. Table 1 The situations encountered by enterprises in production serves as the core input parameter matrix for this program, covering 6 typical production conditions of mechanical component manufacturers. It organizes key indicators across three critical links to support model calculations: the spare part link provides defect rate, purchase price and unit inspection cost for two types of core parts, which matches the supplier selection method in Section 3.1; the finished product link includes defect rate, assembly cost, unit inspection cost and market price, which are essential for profit calculation; the defective finished product handling link details replacement loss and dismantling cost, which aligns with the rework control strategy in Section 3.2. Diverse parameter combinations enable analysis of how parameter changes affect the optimal strategy and total profit.

### 5.2. Numerical Program 2

**Fig 4:**
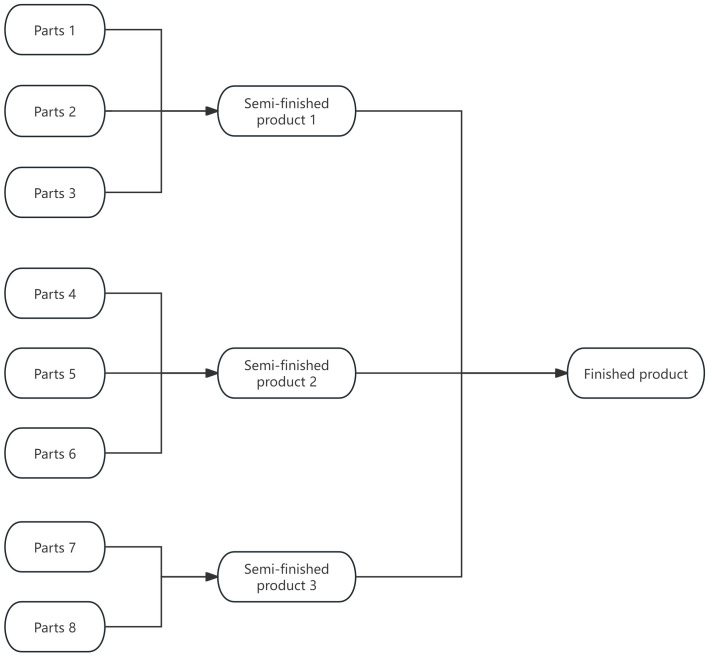
Programming 2 Production flow chart.

This numerical program simulates the more complex “multi-spare part–multi-semi-finished product–finished product” three-stage production process, which is more consistent with the actual operation of mechanical component manufacturers. It is mainly used to verify the adaptability of the joint control strategy in multi-link collaborative scenarios. Fig 4 Programming 2 Production flow chart visually depicts the production process framework: 8 types of spare parts are first grouped into 3 types of semi-finished products, and the three semi-finished products are further assembled into one finished product. The chart also implies key quality control nodes needed in the strategy. Table 2 Situations encountered by enterprises in production provides detailed input parameters corresponding to Fig 4: it includes defect rate, purchase price and unit inspection cost for 8 types of spare parts to support supplier screening in Section 3.1; defect rate, assembly cost and dismantling cost for 3 types of semi-finished products to support rework decisions in Section 3.2; and market price, replacement loss and unit inspection cost for finished products to support the fraction-f sampling plan in Section 3.3. The table covers the full production chain, ensuring authentic simulation and supporting the verification of the joint control strategy in complex scenarios.

### 5.3. Mathematical model solution

**Fig 5:**
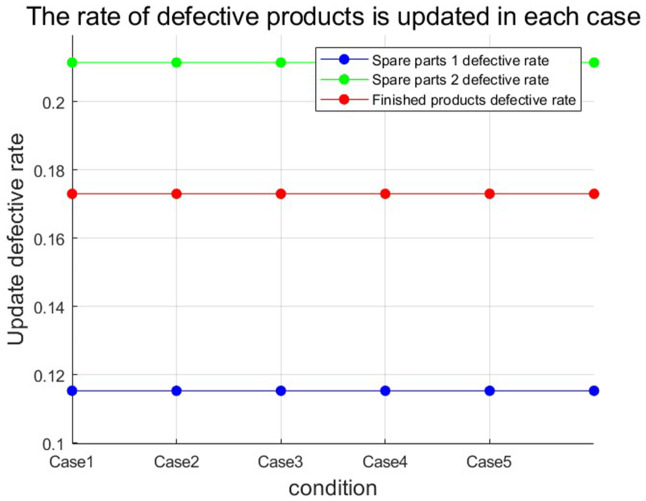
Update of defective rate of numerical program 1.

**Fig 6:**
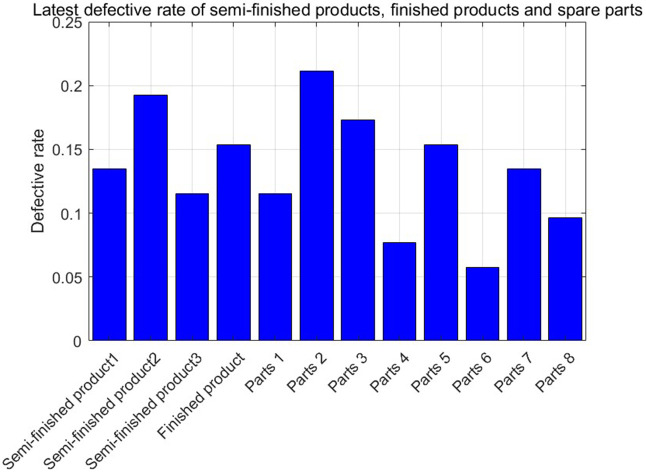
Update of defective rate of numerical program 2.

**Fig 7:**
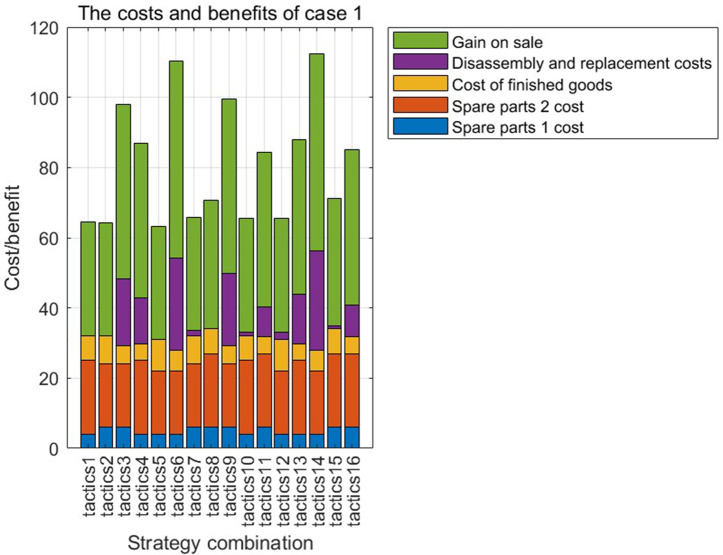
Cost and benefit of each parameter in numerical program 1.

**Fig 8:**
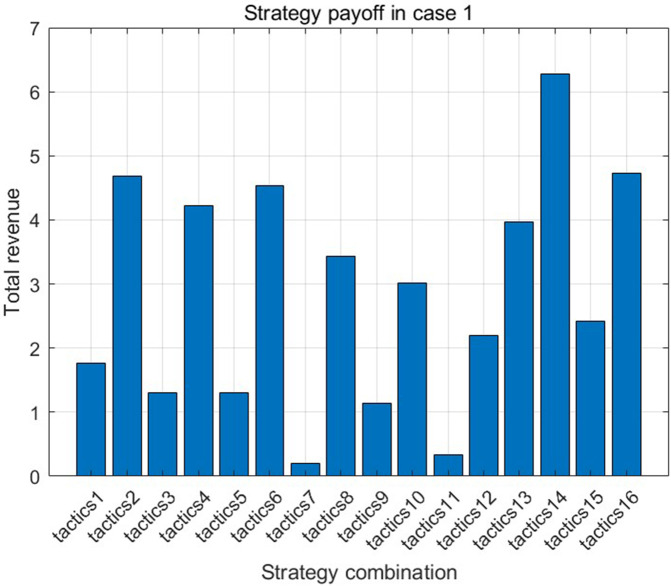
Numerical program 1 Returns of different strategies.

**Fig 9:**
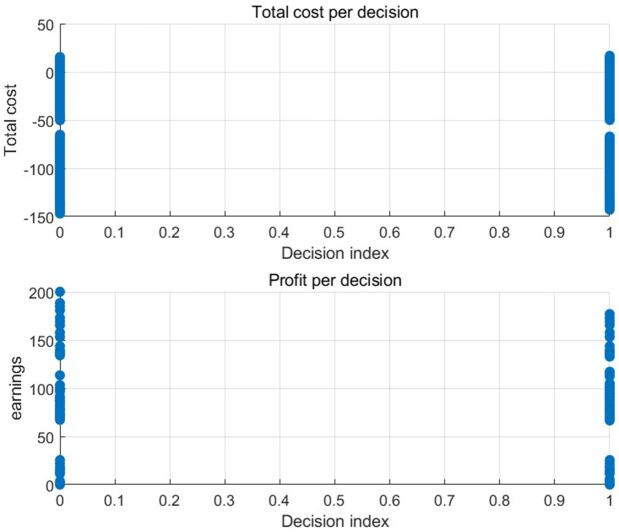
Cost and benefit of each decision in numerical program 2.

**Fig 10:**
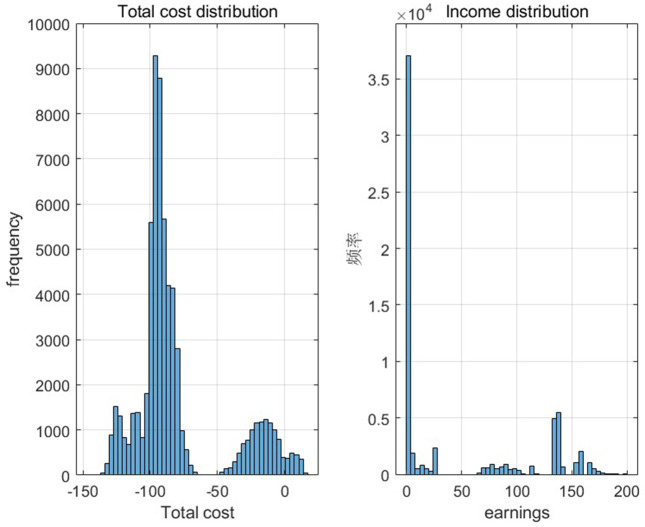
Cost and benefit frequency graph in numerical program 2.

Fig 5 and Fig 6 show the defective rate of each variable after Bayesian updating according to the data given by the numerical program. Table 3 presents the optimal strategy and corresponding gross profit for each case of a finished product in Numerical Program 1. Fig 7 and Fig 8 illustrate cost and benefit distribution of each parameter in Case 1. Table 4 displays the optimal solution for the full complex production process in Numerical Program 2. Fig 9 and Fig 10 depict the cost and profit for each decision variable in Numerical Program 2.

**Table 3:** Optimal scheme and profit for each case in numerical program 1.

Condition	*x*_1_	*x*_2_	*x*_3_	*x*_4_	Gross profit
1	1	1	0	1	6.282
2	1	1	0	1	6.282
3	1	1	1	1	4.735
4	1	1	1	1	8.524
5	0	1	1	1	6.236
6	1	1	0	0	3.476

**Table 4:** Optimal scenarios for each case in numerical program 2.

*x*_1_	*x*_2_	*x*_3_	*x*_4_	*x*_5_	*x*_6_	*x*_7_	*x*_8_
1	0	1	1	0	1	1	0
*x*_9_	*x*_10_	*x*_11_	*x*_12_	*x*_13_	*x*_14_	*x*_15_	*x*_16_
1	1	1	1	1	1	1	1

### 5.4. Case study and multicollinearity analysis

To validate the proposed model, we conduct an empirical study using production data from a precision gear manufacturer in China. Defect rates were estimated via Bayesian updating based on quality inspection records, and cost parameters were obtained from the production accounting system. Table 5 summarizes the parameters, where Part 8 exhibits an identical defect rate of 8.54% to Part 5 and is treated as an outlier and excluded from the core analysis. This exclusion is justified by Part 8’s distinct cost structure (markedly higher purchase and inspection costs) that deviates from the overall distribution of part cost characteristics, which would otherwise introduce statistical bias into parameter estimation and multicollinearity diagnostics.

**Table 5:** Parameter Summary.

Parts	1	2	3	4	5	6	7	8
**Price (CNY)**	45	52	85	78	35	120	18	95
**Inspect (CNY)**	8	8	12	12	6	15	4	10
**Defect rate**	10.98%	9.76%	15.85%	12.20%	8.54%	6.10%	7.32%	8.54%
**Semi-finished**	1		2		3		**Finished**	
**Assembly (CNY)**	65		85		45		120	
**Inspect (CNY)**	25		30		20		45	
**Disassembly (CNY)**	45		55		35		85	
**Defect rate**	9.76%		12.20%		7.32%		10.98%	
**Selling price (CNY)**	2850							
**Replacement loss (CNY)**	580							

The model was solved by enumerating all decision combinations. The optimal strategy inspects parts 1–6 while skipping parts 7–8 with lower defect rates, inspects and disassembles all defective semi-finished products, and performs final inspection with disassembly. This achieves a unit profit of 1,046.06 CNY compared to 604.20 CNY under current practice, yielding a 73.13% improvement.

To ensure the robustness of these results, we examined potential multicollinearity among model parameters using Latin Hypercube Sampling with ±30% variation. Variance Inflation Factors and condition numbers were computed from the regression of optimal profit on parameter values, as shown in Table 6.

**Table 6:** Multicollinearity Diagnostics.

Parameter Group	Mean VIF	Max VIF	Status
Part defect rates	1.15	1.26	Good
Semi/Finished defect rates	1.13	1.17	Good
Part costs	1.13	1.19	Good
Assembly costs	1.20	1.30	Good
Condition Number	1.97		< 30
R²	0.9243		High

All VIF values are below 1.30, well under the threshold of 5, and the condition number of 1.97 confirms no severe multicollinearity among the model’s covariate predictors, which include part defect rates, semi-finished product defect rates, finished product defect rates, part purchase costs, part inspection costs, semi-finished product assembly costs, semi-finished product inspection costs, semi-finished product disassembly costs, finished product assembly costs, finished product inspection costs, finished product disassembly costs, and finished product replacement losses. The R² of 0.9243 indicates strong explanatory power.

## 6. Sensitivity analysis

In order to deeply explore the effect of the defective rate on the optimal solution, a systematic sensitivity analysis is carried out with the aid of data program 1 and data program 2.

### 6.1. Data program 1 Analyze the results

**Fig 11:**
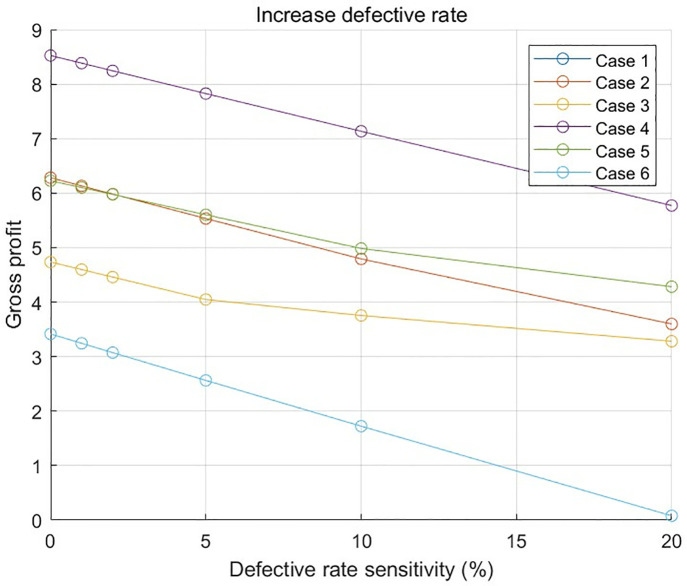
Relationship between the increase of defective parts rate and profit in numerical program 1.

**Fig 12:**
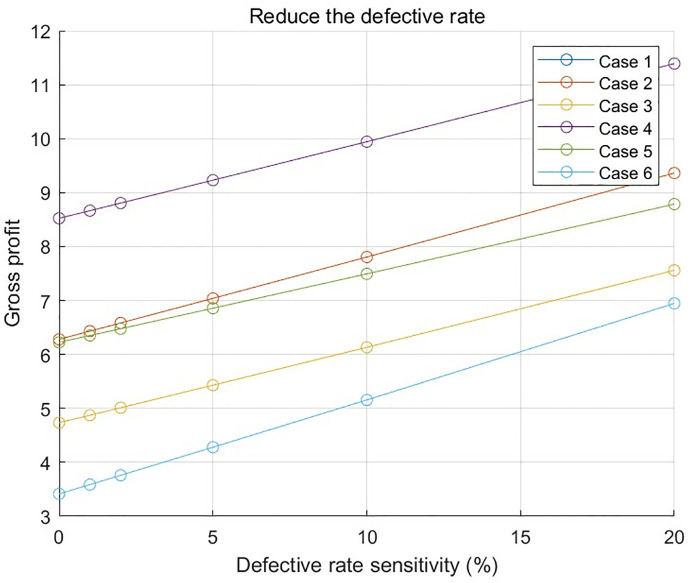
Relationship between the reduction of defective parts rate and profit in numerical program 1.

Fig 11 and Fig 12 show the relationship between the profit and a 20% increase, as well as a 20% decrease, in the part defect rate in each case in numerical program 1, respectively. It can be clearly observed from the Fig that the impact of the defective parts rate on the total profit shows a significant difference under the complex situation of considering the combination of many different factors, such as the defective parts rate, the inspection cost of parts 1, the inspection cost of parts 2, the inspection cost of finished products, and the replacement loss and dismantling cost of unqualified finished products. This result verifies the accuracy of the model in evaluating the impact of the defective rate on the final result and further confirming the rationality and validity of the model.

### 6.2. Data Program 2 Analysis results

**Fig 13:**
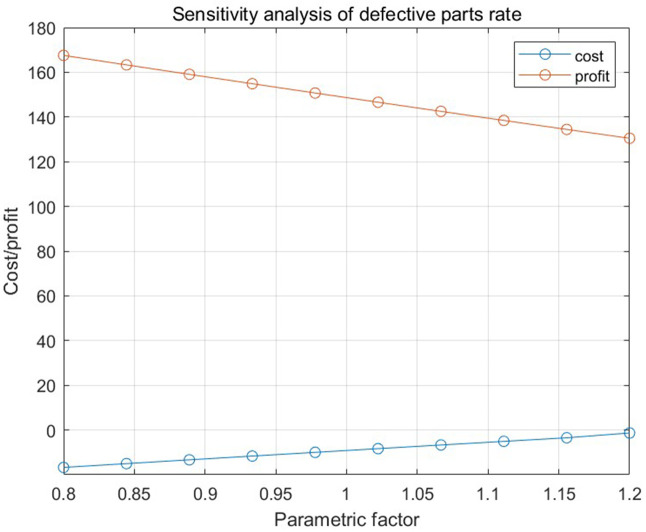
The relationship between the defective rate of parts and the total cost and total profit in numerical program 2.

**Fig 14:**
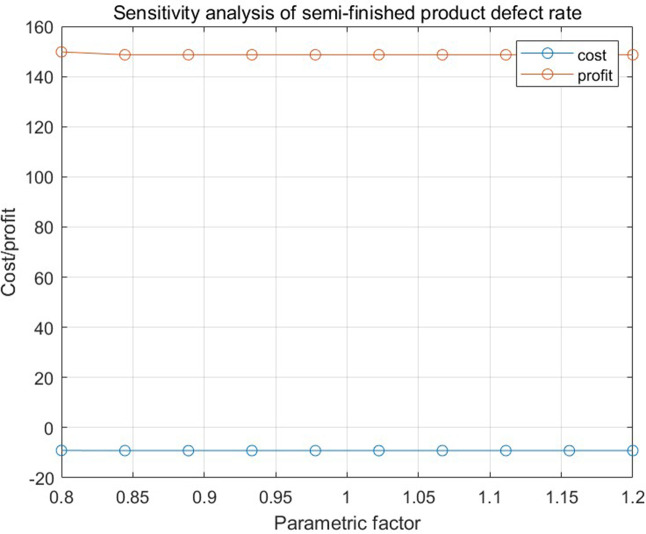
The relationship between the defective rate of semi-finished products and the total cost and total profit in numerical program 2.

**Fig 15:**
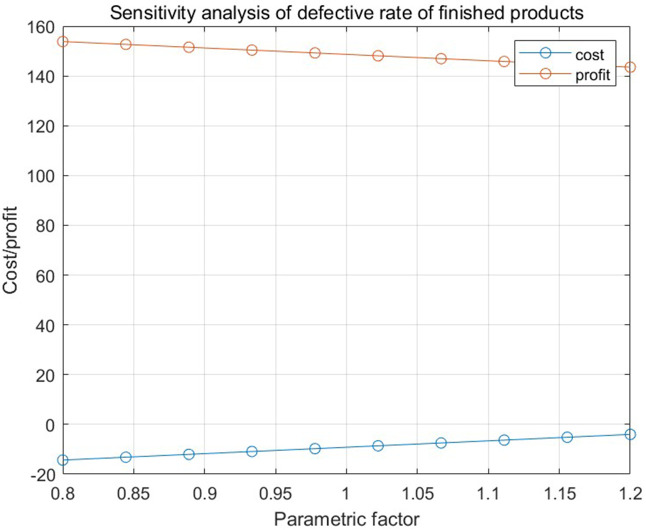
The relationship between the defective rate of finished products and the total cost and total profit in numerical program 2.

Fig 13, Fig 14, and Fig 15, respectively, show the impact of the defective rate on the total profit under the combination scenario of 8 types of parts, 3 types of semi-finished products, 1 finished product inspection cost, and replacement loss and dismantling cost of unqualified finished products in numerical program 2 when the adjustment range of the defective rate of parts, semi-finished products, and finished products is 20%. The results show that different factors have different degrees of influence on total profit.

These sensitivity analysis charts provide valuable decision-making basis for enterprises, help enterprises to identify and focus on the factors that have a greater impact on profits, and then adopt targeted measures to optimise the cost structure and enhance the profit level.

## 7. Cost efficiency comparative analysis

In this section, we conduct a comparative analysis of the proposed integrated policy and the existing alternatives to demonstrate its superior performance in terms of cost.

Five alternative strategies were considered, using the data and conditions in Numerical Program 2. Each strategy optimized joint production, maintenance, and quality control, and each strategy adopted different inspection strategies and error models. The definitions of these strategies are as follows:

(1) Recommended Strategy: Adopt the optimized collaborative control strategy, combined with sampling inspection, to balance cost and quality targets.
(2) Inspection @ 0%: This strategy does not conduct any inspection; all products directly enter the production or sales process, suitable for scenarios with cost sensitivity but lower quality requirements.
(3) Inspection @ 100%: This strategy uses 100% inspection to ensure that each product is inspected, suitable for production environments with high quality requirements or for critical products.
(4) AQL (Acceptable Quality Level): Set the producer’s acceptable upper limit at 10% defect rate, through sampling inspection to balance cost and quality in large-scale production.
(5) LTPD (Lot Tolerance Percent Defective): Set the consumer risk control threshold at 10% defect rate, focusing on reducing the probability of defective products entering the market.

In product quality management, LTPD (Lot Tolerance Percent Defective) and AQL (Acceptable Quality Level) are two sampling inspection strategies, serving consumers and producers respectively. LTPD sets a higher defect rate limit to ensure that the proportion of defective products for consumers does not exceed this threshold, thereby reducing consumer risks and making it suitable for high-reliability scenarios; AQL defines the acceptable defect rate from the producer’s perspective, through sampling to balance cost and quality, suitable for large-scale production. Both use limited sampling to assess batch quality. LTPD focuses on consumer protection, while AQL pays attention to producer needs.

For each strategy, Table 7 presents the performance data of five quality control strategies, including the recommended strategy, Inspection @ 0%, Inspection @ 100%, AQL, and LTPD, covering key indicators such as total profit, average outgoing quality (AOQ), inspection cost, and defect rate. The recommended strategy stands out with excellent performance of total profit 16.6768 and AOQ 0.0189, demonstrating its dual advantages in economic benefits and quality control. In contrast, Inspection @ 0% without any inspection leads to a high defect rate of 0.1000, an AOQ of 0.1330, and a sharp drop in total profit to −110.6495, reflecting the severe consequences of quality out-of-control. Inspection @ 100% controls the AOQ at 0.0181 through 100% inspection, but the inspection cost is as high as 20.4615, and the profit is only 15.1943, with insufficient cost performance. AQL and LTPD have profits of 14.1578 and 15.1861, and AOQs of 0.0390 and 0.0425, respectively, showing stable performance but not reaching the optimal level. The table clearly shows that the recommended strategy achieves a better balance in multi-dimensional indicators.

**Table 7:** Strategy Comparison.

policies	$p_{z}$	AOQ	$C_{j}$	$N_{2}$	producton cost
Propose Policy	16.6768	0.0189	20.2297	0.0357	114.8097
Inspection @ 0%	−110.6495	0.1330	0.0000	0.1000	347.9747
Inspection@ 100%	15.1943	0.0181	20.4615	0.0050	137.5948
AQL	14.1578	0.0390	19.0001	0.0950	137.3329
LTPD	15.1861	0.0425	17.4615	0.0964	137.5948

The analysis shows that the recommended strategy outperforms other strategies in total profit (16.6768), quality (AOQ 0.0189), and cost control (inspection cost 20.2297) through dynamic optimization of the inspection process. The defect rate is as low as 0.0050, demonstrating efficient quality management capabilities. Inspection @ 0% suffers from the lack of any quality intervention, with production costs soaring to 347.9747 and a severe loss in profit, proving the necessity of inspection. Inspection @ 100% although strict in quality control, has an excessively high inspection cost, undermines economic efficiency, resulting in lower profit than that of the recommended strategy. AQL and LTPD operate with a 10% defect rate standard, resulting in high defect rates (0.0950 and 0.0964) and limited quality control effects, leading to lower profits. The reason why the recommended strategy is superior is that it combines dynamic adjustment and defect modeling, enabling it to adapt to changes in the production system, maximizing profits while ensuring high quality, demonstrating its significant advantages in practical manufacturing applications.

## 8. Conclusion

This study addresses the collaborative optimization of procurement, production, rework, and quality control in mechanical component manufacturing enterprises—a sector characterized by stringent precision requirements and high sensitivity to raw material cost fluctuations. To address key limitations in prior research—including fragmented single-stage approaches and inadequate attention to the interplay between inspection errors and production system dynamics—this study introduces an innovative joint control strategy supported by a multi-link mathematical model. The proposed strategy integrates three core components: supplier screening based on 90% confidence intervals and binomial distribution-based sample sizes, dynamic rework decisions through cost-benefit analysis, and fraction-f sampling for quality control. By incorporating Type I and Type II inspection errors (producer and consumer risks) and production system errors (source and chain errors), the framework enables cross-stage cost-quality optimization tailored to the specific requirements of mechanical component manufacturing realities.The sensitivity analysis yields critical insights: ± 20% variations in spare part defect rates result in substantial profit shifts, thereby underscoring procurement quality as a pivotal efficiency driver; semi-finished product defect rates exert minimal influence, confirming the efficacy of in-process control efficacy; and elevated finished product defect rates amplify compensation and replacement costs, highlighting the importance of rigorous final inspections and post-sales management.

The unique contributions of this study stem from both its industry alignment and methodological rigor, and can be summarized as follows: (1) This study develops a sector-specific multi-stage model tailored to mechanical component manufacturing complexities, which unifies supplier screening (with statistical sampling for defect control and error assessment), semi-finished product disassembly (based on revenue-cost parity), and dynamic quality sampling—thereby addressing gaps in prior studies that neglect inspection error-system the interdependencies between inspection errors and production systems; (2) It validates the model with 2023 industry data on operational indicators from 15 representative enterprises in key Chinese regions, enhancing real-world applicability, while employing Bayesian updating to overcome static parameter constraints in traditional models and enable real-time defect rate adaptation; (3) The study quantifies the defect rate-profit relationships through sensitivity analysis, thereby identifying stage-specific impacts and providing actionable benchmarks for enterprises; (4) The proposed strategy demonstrates superiority over alternatives (0% inspection, 100% inspection, AQL, LTPD), achieving balanced outcomes: profit of 16.6768, AOQ of 0.0189, and inspection cost of 20.2297—surpassing 100% inspection (profit: 15.1943, cost: 20.4615) economically and AQL/LTPD (AOQ: 0.0390/LTPD: 0.0425) in quality, while averting 0% inspection losses (profit: −110.6495, AOQ: 0.1330).

Theoretically, this research advances operations management by extending multi-stage optimization to mechanical component manufacturing with its precision and cost constraints; integrating inspection and system errors into a cohesive framework, surpassing fuzzy optimization-focused studies; and replacing rigid AQL/LTPD plans with adaptive fraction-f sampling responsive to inspection costs and AOQ needs. From a managerial perspective, this study equips enterprises with practical tools: Eq. 19’s confidence interval model accepts supplier batches only if the defect rate upper limit does not exceed the nominal rate; Eqs. 5–6 guide cost-effective semi-finished product disassembly; and the fraction-f plan optimizes quality-cost trade-offs compared to full or zero inspections. Furthermore, the MATLAB simulations developed for two- and three-stage scenarios provide a platform for data-driven scenario testing and parameter calibration.

Despite these contributions, several limitations of this study should be acknowledged: (1) The model simplifies supply chain network structures by focusing on single-finished-product scenarios, without accounting for multi-product co-production complexities that are common in actual mechanical component manufacturing, potentially limiting its adaptability to enterprises with multi-product lines; (2) Certain operational parameters in the model are calibrated based on industry average values derived from the dataset, which may not fully reflect individual differences in cost structures and operational efficiencies among enterprises of varying sizes; (3) The model assumes stable raw material supply and fixed purchase prices, thereby excluding the impact of raw material price volatility (a key challenge for mechanical component manufacturing) on procurement costs and defect rate-related decisions. Future research will address these limitations by: (1) Extending the model to multi-product co-production scenarios, integrating shared resource scheduling logic to enhance adaptability to complex production lines; (2) Introducing enterprise-scale classification variables to calibrate parameters based on enterprise size and type, thereby improving the model’s applicability across for different operational entities; (3) Incorporating raw material price fluctuation models into the procurement module to optimize supplier selection and order quantity determination under conditions of price uncertainty; (4) Developing decision support systems to facilitate real-time, data-driven decision-making; (5) Adopting online inspection technologies to improve real-time monitoring and response capabilities; (6) Integrating predictive maintenance technologies to mitigate equipment-related inspection errors and strengthen supply chain resilience. In conclusion, the proposed joint control strategy provides a rigorous and practical solution for mechanical component manufacturing, effectively bridging the gap between theoretical modeling and industrial application while optimizing cost, quality, and operational efficiency.

## Supporting information

S1 File Supplemental Data for Numerical Programs and Sensitivity Analysis. This File contains the defect rate data updated using Bayesian methods, the output results of two numerical optimization programs, and the raw sensitivity analysis data for each numerical program. All data are presented in Excel tables and are provided to validate and reproduce the numerical methods and sensitivity analysis process used in the study.
(RAR)

S2 File MATLAB Code for Computational Results and Visualization. This File includes the MATLAB code used for all the computational results in the study, including the outputs of sensitivity analysis, optimization programs, and the corresponding code for generating figures. Users can utilize this code to reproduce the data processing, numerical calculations, and visualizations, with the generated figures helping to further understand the methods and results used in the study.
(ZIP)
